# *Brucella melitensis* Methionyl-tRNA-Synthetase (MetRS), a Potential Drug Target for Brucellosis

**DOI:** 10.1371/journal.pone.0160350

**Published:** 2016-08-08

**Authors:** Kayode K. Ojo, Ranae M. Ranade, Zhongsheng Zhang, David M. Dranow, Janette B. Myers, Ryan Choi, Steve Nakazawa Hewitt, Thomas E. Edwards, Douglas R. Davies, Donald Lorimer, Stephen M. Boyle, Lynn K. Barrett, Frederick S. Buckner, Erkang Fan, Wesley C. Van Voorhis

**Affiliations:** 1 Center for Emerging and Re-emerging Infectious Diseases (CERID), Department of Medicine, Division of Allergy and Infectious Diseases, University of Washington, Seattle, Washington, United States of America; 2 Department of Biochemistry, University of Washington, Seattle, Washington, United States of America; 3 Beryllium, Bainbridge Island, Washington, United States of America; 4 Center for Molecular Medicine and Infectious Diseases, Department of Biomedical Sciences and Pathobiology, Virginia Polytechnic Institute and State University, Blacksburg, Virginia, United States of America; Universidad de Santiago de Compostela, SPAIN

## Abstract

We investigated *Brucella melitensis* methionyl-tRNA-synthetase (*Bm*MetRS) with molecular, structural and phenotypic methods to learn if *Bm*MetRS is a promising target for brucellosis drug development. Recombinant *Bm*MetRS was expressed, purified from wild type *Brucella melitensis* biovar *Abortus* 2308 strain ATCC/CRP #DD-156 and screened by a thermal melt assay against a focused library of one hundred previously classified methionyl-tRNA-synthetase inhibitors of the blood stage form of *Trypanosoma brucei*. Three compounds showed appreciable shift of denaturation temperature and were selected for further studies on inhibition of the recombinant enzyme activity and cell viability against wild type *B*. *melitensis* strain 16M. *Bm*MetRS protein complexed with these three inhibitors resolved into three-dimensional crystal structures and was analyzed. All three selected methionyl-tRNA-synthetase compounds inhibit recombinant *Bm*MetRS enzymatic functions in an aminoacylation assay at varying concentrations. Furthermore, growth inhibition of *B*. *melitensis* strain 16M by the compounds was shown. Inhibitor-*Bm*MetRS crystal structure models were used to illustrate the molecular basis of the enzyme inhibition. Our current data suggests that *Bm*MetRS is a promising target for brucellosis drug development. However, further studies are needed to optimize lead compound potency, efficacy and safety as well as determine the pharmacokinetics, optimal dosage, and duration for effective treatment.

## Introduction

*Brucella* spp., small facultative intracellular coccobacilli, function as facultative intracellular bacteria causing the zoonotic disease, brucellosis. These bacteria are transmitted from animals to humans by ingestion of infected food products, direct contact with tissues of an infected animal, or inhalation of aerosols. Although humans are considered accidental hosts, brucellosis remains a worldwide public health concern [1]. The effects of human brucellosis can range from an acute febrile disease to persistent neurological disorders, osteomyelitis, endocarditis, and other syndromes[2]. Eight species have been identified, and of these, 4 species—*B*. *abortus*, *B*. *canis*, *B*. *suis* and *B*. *melitensis—*infect livestock and have moderate to high human pathogenicity [1, 3]. While brucellosis remains a zoonotic disease, it can also be transmitted by unpasteurized milk products from various species. Today’s global burden of human brucellosis remains enormous, with more than 500,000 human infections per year worldwide [4]. Furthermore, *Brucella* spp. exemplify an ever present threat as potential bioterrorism weapons [5], which underscores the need to validate novel drug targets for new therapeutics. *Brucella* species pose a significant public health concern by the rise of antimicrobial resistance and their potential exposure to sub-therapeutic antibiotics in livestock and humans. Brucellosis remains a very difficult disease to treat due to the bacteria’s ability to reside for extended periods inside the host's cells through evasion of the immune response and inhibition of programmed cell death [6, 7]. The relative lack of efficacy of standard antibiotics on this intracellular bacterial pathogen also affects successful treatment. Current therapeutic options in order to avoid relapses and to prevent prolonged use of these drugs include combinations of the antibiotics: doxycycline, rifampin and streptomycin [8, 9]. High incidences of therapeutic failures (relapses) have been observed even with prolonged treatment regimens, possibly due to resurgence and outgrowth of intracellular reservoirs of *Brucella*. However, therapeutic failures may also be due to *Brucella* antibiotic resistance associated with increasing prevalence of drug-resistance genes for the brucellosis first-line treatment options [10, 11].

Hence, it remains a high priority to develop inexpensive, non-toxic, orally available brucellosis therapeutics utilizing new mechanisms of drug action. Advances in molecular biology and the availability of full genome sequences of *Brucella* species have increased the prospects for discovering druggable enzyme targets by exploiting the biochemical and physiological differences between pathogen and host. Selective disruptions of microbial protein translation processes have been successfully exploited in different classes of antimicrobial therapies. The aminoacyl-tRNA synthetases (aaRSs) are among the essential enzymes in cell protein translation processes and are generating increased interest from a drug development standpoint [12]. A number of natural antimicrobials have been shown to specifically inhibit aaRSs, validating these as drug targets [13].

Within the twenty aaRSs, methionyl-tRNA synthetase (MetRS) is especially interesting for it not only links tRNA with methionine for elongation in protein synthesis, but also links the initiator tRNA with methionine for protein synthesis [14]. Previously solved crystal structures of bacterial MetRSs yield interesting insights into both enzyme architecture and methionylation catalysis. MetRS occurs in two major forms, MetRS1 and MetRS2. They can be distinguished based on amino acid sequence similarity and the presence of a number of zinc knuckle domains [15, 16]. Specific inhibitors of MetRS1 have been shown to be potential drugs for treatment against the bacterial pathogens: *Staphylococcus aureus* [17] and *Clostridium difficile* [18]while MetRS2 inhibitors of *Escherichia coli* methionyl-tRNA synthetase (*Ec*MetRS) have been described [19]. Sensitivity to inhibition by individual small molecule scaffolds seems to vary according to the specific MetRS form. We have demonstrated that analogs of MetRS1 inhibitors for *S*. *aureus* and *C*. *difficile* can selectively target *Trypanosoma brucei* MetRS (*Tb*MetRS) with potent therapeutic activity in the mouse model of trypanosomiasis [12, 20]. We have also shown that pharmacological properties of these series of inhibitors could be greatly improved using structure-based drug design [12, 20]. In this report, we describe biochemical, structural, and inhibitory studies of *B*. *melitensis* methionyl-tRNA synthetase (*Bm*MetRS) and inhibitor activity against *B*. *melitensis*. Selective inhibitors from the *Tb*MetRS series inhibited both the function of recombinant *Bm*MetRS and the growth of *Brucella in vitro*. Optimization of lead candidates for development of potent therapeutics requires detailed understanding of inhibitor structural-activity relationships (SAR). X-ray crystal structures could effectively guide medicinal chemistry by predicting alterations and functional groups needed to improve potency, selectivity, and pharmacokinetic properties. The crystal structure of *Bm*MetRS was solved in complex with selenomethionine and with three different inhibitors to define structural pockets that can be further exploited for the development of potent lead candidates.

## Materials and Methods

### Compound library

Methionyl-tRNA synthetase inhibitors used in this study were previously described [12, 20, 21]. Chemical purity of all compounds (>98%) was confirmed by reverse-phase HPLC and ^1^H-NMR then reconstituted and stored at -20°C in 100% DMSO at a final concentration of 20 mM.

### Molecular cloning, protein expression and purification of *Brucella* MetRS

The complete coding region of methionyl-tRNA synthetase was PCR amplified from genomic DNA extracted from wild type *Brucella melitensis* biovar *Abortus* 2308 strain ATCC/CRP #DD-156. Sequence similarities and conserved domain alignments of various *Brucella* spp. methionyl-tRNA synthetase open reading frames were performed using the online tools (BLAST and CDART) available at the National Center for Biotechnology Information (http://blast.ncbi.nlm.nih.gov/Blast.cgi). Because the available *Brucella* species MetRS gene sequences seem to be highly conserved, we do not anticipate any structural differences or response to inhibitors between strains or species. The amplicons were cloned into the ligation independent cloning (LIC) site of plasmid expression vector AVA0421 [22, 23]. Inserts were sequenced for confirmation with GenBank entries. Recombinant expression was in Rosetta^®^ 2(DE3) competent *E*. *coli* (Novagen EMD, Billerica, MA) using Studier auto-induction protocols at 20°C [24]. Soluble enzymes were purified by immobilized metal-affinity chromatography (IMAC) in a Ni^2+^-NTA (Qiagen, Valencia, CA) column followed by size exclusion chromatography in a 26/60 Superdex 75 SEC column as earlier described [25]. The binding buffer was composed of 20 mM HEPES pH 7.25, 500 mM NaCl, 5% glycerol, 30 mM imidazole, 0.5% CHAPS, and 1 mM TCEP. Purified proteins were eluted in the same buffer supplemented with 250 mM imidazole.

### Thermal shift assay

Thermal stability of recombinant *Bm*MetRS in the presence or absence of inhibitors was determined in a 96-well format as previously described [26]. Each assay well was composed of 0.16 mg/mL *Bm*MetRS enzyme, 0.1 mM of each inhibitor when present, and 5% dimethyl sulfoxide (DMSO). All assays were performed independently three times.

### Aminoacylation assays

Enzyme activity was quantified by the attachment of [^3^H]-L- methionine to tRNA in the presence of *Bm*MetRS enzymes as previously described [12]. All assays were run in 96-well filter plates with Durapore membranes (Millipore, Billerica, MA) in a final reaction volume of 75 μL. Enzyme activity assays were performed in the presence of 25 mM HEPES-KOH, pH 7.9, 10 mM MgCl_2_, 50 mM KCl, 0.2 mM spermine, 0.1 mg/mL bovine serum albumin, 2.5 mM dithiothreitol, 0.1 mM ATP, 240 nM [^3^H]-L- methionine (83 Ci/mmol), and 5.3 U/mL pyrophosphatase (Sigma, St. Louis, MO). Assays were performed to determine which tRNA source gave better activity with *Bm*MetRS, bulk brewer’s yeast tRNA (Roche Life Sciences, Indianapolis, IN) or bulk *Escherichia coli* tRNA (Sigma), and the bulk brewer’s yeast tRNA was found to be superior. This preliminary reaction was determined in the presence of 100 nM recombinant *Bm*MetRS and serial two-fold dilutions of the tRNAs from 400 to 6.25 μg/mL. Enzyme reaction progress time curve was determined at 0, 15, 30, 60, 120 and 180 minutes with two fold serial concentrations (0, 1.5, 3, 6, 12 nM) of *Bm*MetRS using 400 μg/mL bulk brewer’s yeast tRNA. Inhibition of aminoacylation by synthesized compounds was assayed in the buffered medium, except the amount of pyrophosphatase was reduced to 0.1 U/mL to conserve this reagent, using 2 nM recombinant *Bm*MetRS pre-incubated with 10 μM to 0.056 nM serial dilutions of inhibitors into a total volume of 75 μL. The reaction was initiated by the addition of 400 μg/mL bulk brewer’s yeast tRNA. The plate was incubated without shaking at room temperature for 120 min. Reactions were terminated by the addition of 100 μL cold 10% trichloroacetic acid (TCA). Using a vacuum manifold the precipitated tRNA-[^3^H]-L-methionine product was transferred to Durapore membranes plates (Millipore), which were washed three times with cold 10% TCA and dried. The reaction was read on a MicroBeta2^®^ plate reader (PerkinElmer, Waltham, MA). Samples were run in triplicate, and the average activity of inhibitors was compared to that in control wells without inhibitors [12].

### Bacterial strain and growth conditions

All *Brucella* growth experiments were performed in a BSL-3 laboratory located in the Virginia-Maryland Regional College of Veterinary Medicine, Virginia Tech, Blacksburg, VA, USA and certified annually by the U.S. Centers for Disease Control and Prevention. *Brucella melitensis* 16M, wild type and fully virulent, was obtained from the culture collection (Veterinary Medicine, Virginia Tech) and grown in either liquid minimal medium [27] or on minimal medium agar plates at 37°C in 5% CO_2_. A working plate stock (WPS) was prepared by inoculation of an aliquot of a -80°C frozen strain onto a minimal agar plate and grown 3–4 days; the WPS was then stored at 4°C for up to one month. One or two colonies were picked from the WPS and used to inoculate 25 ml of minimal medium and shaken at 180 rpm in a rotary air incubator until the culture reached late log phase, OD_450_ = 0.8.

### Minimum inhibitory concentration determination

A late log phase culture was diluted 1:10 (OD_450_ = 0.05–0.1) in minimal medium and incubated with indicated serial concentrations of inhibitors (total volume = 100 μL) in triplicates using a 96-well, low adhesion 3474 microtiter plate (Corning Inc., Corning, NY) [28]. Positive and negative controls included in each plate comprised of 50 μg/mL gentamicin and DMSO respectively. Gentamicin was used as a positive control as it is routinely bactericidal to *Brucella* spp. The mechanisms of gentamicin and MetRS inhibitors are similar. Gentamicin inhibits protein synthesis at the level of the 30S ribosomal subunit. To reduce edge effects and evaporation, the wells on the entire perimeter of the plate were not used for growth, but had 200 μL of minimal medium added to help reduce evaporation. The plates were sealed with an adhesive Microseal B lid (Bio-Rad, Hercules, CA) and incubated at 37°C in a VersaMax Tunable Microplate Reader (Molecular Devices, Sunnyvale, CA) at the following settings: Kinetic, 450 nm; before readings at 28 hours, plates were shaken for 10 sec., read every 15 min., reduction set at 4. The lowest concentration of an inhibitor required to inhibit the growth of *B*. *melitensis* strain 16M (MIC) was performed by a standard method [28]. Subsequent analysis and calculation of inhibitors MIC values were performed using Graphpad^®^ Prism software (GraphPad Software, San Diego, CA).

### Protein crystallization

Purified recombinant *Bm*MetRS was concentrated to 20 mg/mL and incubated with either 5 mM selenomethionine or ATP, 2 mM **1312**, 3 mM **1415**, or 3 mM **1433** for 5 minutes at 289 K. For all complexes, crystals were then grown at 289 K by sitting drop vapor diffusion with 0.4 μL of protein/ligand complex mixed with 0.4 μL reservoir solution equilibrated against 80 μL of reservoir solution. Each reservoir solution differed: for selenomethionine crystals, the reservoir solution was 0.2 M ammonium nitrate, 20% PEG-3350, pH = 7.5; for **1312** crystals, the reservoir solution was 25% PEG-1500, 100 mM MMT buffer, pH = 5.0; for **1415** crystals, the reservoir solution was 25% PEG-1500, 7% 10x PCB, pH = 4.0, 3% 10x PCB, pH = 9.0; and for **1433** crystals, the reservoir solution was 0.2 M ammonium acetate, 0.1 M sodium acetate trihydrate, pH = 4.6, 30% PEG-4000. All crystals were cryo-protected in a solution containing 80% reservoir solution and 20% ethylene glycol.

### X-ray diffraction and structure determination

Data for *Bm*MetRS with selenomethionine were collected at 100 K on a Rayonix MX-225 detector at a wavelength of 0.9787 Å on beamline 21-ID-F at the Advanced Photon Source (APS, Argonne, IL). Data for *Bm*MetRS with **1312** were collected at 100 K on an ADSC Quantum 210R detector at a wavelength of 0.95370 Å on beamline MX1 at the Australian Synchrotron (AUS, Clayton, Victoria). Data for *Bm*MetRS with **1415** were collected at 100 K on a Rayonix MX-225 detector at a wavelength of 0.9786 Å on beamline 21-ID-F at the Advanced Photon Source (APS). Data for *Bm*MetRS with **1433** were collected at 100 K on a Rayonix MX-300 detector at a wavelength of 0.9786 Å on beamline 21-ID-G at the Advanced Photon Source (APS). For all datasets, indexing and integration were carried out using XDS and the scaling of the intensity data was accomplished with XSCALE [29]. For *Bm*MetRS with selenomethionine, the structure was solved using molecular replacement with Phaser [30] with 2X1L as a starting model. All subsequent structures were solved using Fourier synthesis with Refmac5 [31] with the *Bm*MetRS with selenomethionine structure providing the phases. For all structures except the **1312** structure, refinement was carried out using Refmac5, TLS [32] and Coot [33]. For the **1312** structure, the final refinement was carried out with Phenix [34]. All structures were quality checked by Molprobity [35].

## Results

### Activity of aminoquinolone and urea-based inhibitors in thermal stability assay and inhibition of *Bm*MetRS in enzyme activity assay

**Fig 1:**
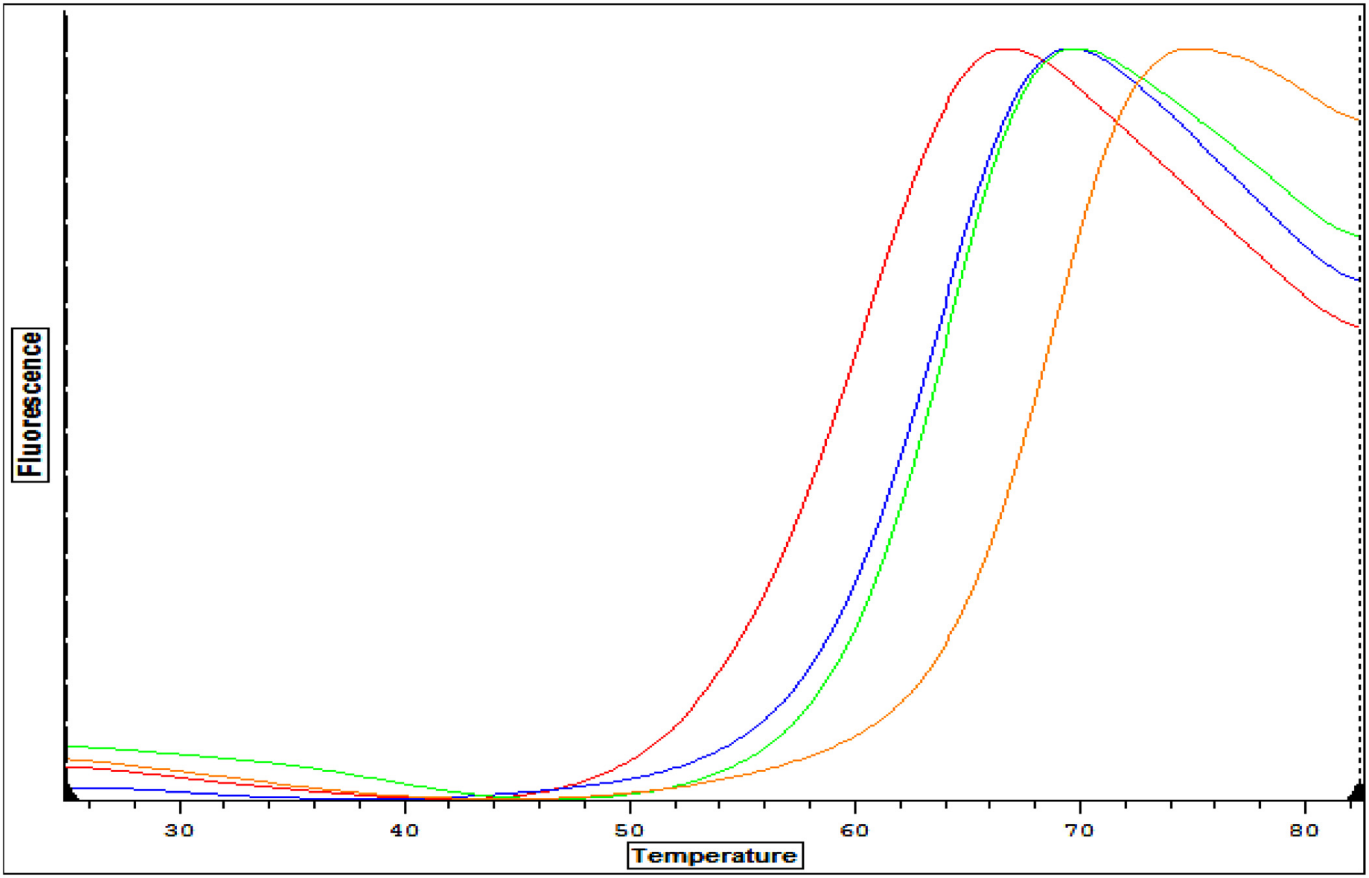
Comparative analysis of thermal stability plots of recombinant *Bm*MetRS with ligands. Thermal shift assays T_m_ curves for apo form (control) of the *Bm*MetRS (red plot) and inhibitors bound with **1312** (amber), **1433** (blue) and **1415** (green) plots. A significant shift in melting temperature (ΔT_m_) of 7.8°C was observed when compound **1312** was complexed with *Bm*MetRS compared to the Apo *Bm*MetRS (unbound). Two other compounds, **1415** and **1433**, exhibited Δ*T*_m_s of 3.1°C and 2.5°C respectively.

A set of 100 aminoquinolone and urea-based inhibitors were screened by thermal shift assays as an indicator of binding affinity to *Bm*MetRS [12, 20]. The melting temperature (T_m_) of Apo (unbound) *Bm*MetRS was 60.6°C. A significant temperature shift (ΔT_m_) of 7.8°C was observed for compound **1312**. We also identified 2 other compounds, **1415** and **1433**, exhibiting Δ*T*_m_s of 3.1°C and 2.5°C respectively (Fig 1).

**Fig 2:**
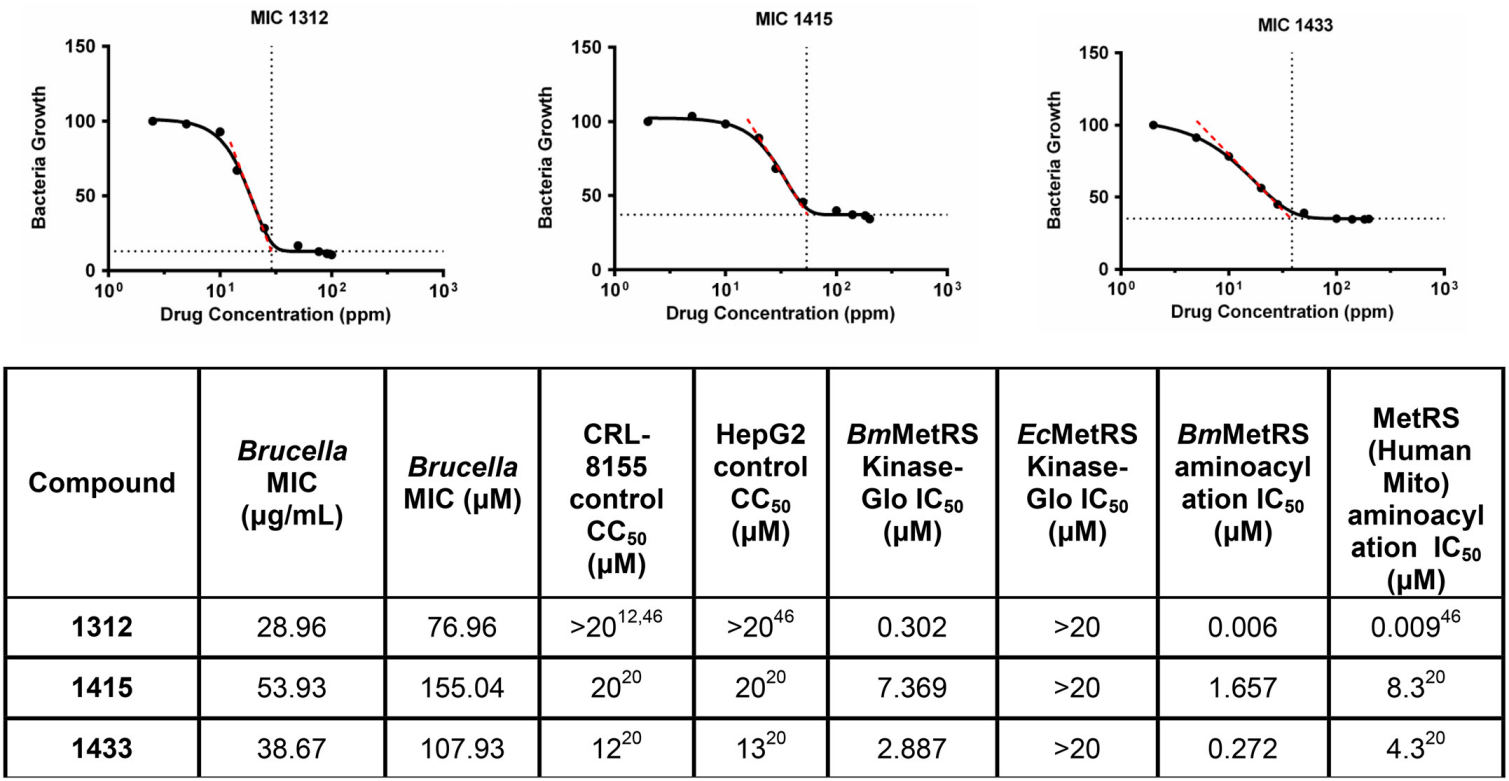
Growth inhibition. The lowest effective concentration of a methionyl-tRNA synthetase inhibitor required to inhibit the growth of *B*. *melitensis* strain 16M (MIC) was assessed from values observed over concentrations ranging from 0.716–71.6 μg/mL for compound **1433,** 0.696–69.6 μg/mL for **1415** while **1312** was determined at inhibitor concentration 0.865–37.6 μg/mL. Average growth (OD_450_ nm) at 28 hours (late log phase), 37°C, in *Brucella* minimal medium was measured. Inhibitor **1415** MIC = 53.93μg/mL; Inhibitor **1433** MIC = 38.67 μg/mL; Inhibitor **1312**, MIC = 28.96 μg/mL. Control = gentamicin, no growth, 0.7–50 μg/mL; DMSO control has no inhibition of bacterial growth. Data table shows MICs from *B*. *melitensis* growth inhibition and IC_50_s of inhibitors (Kinase-Glo^®^, aminoacylation assays). The characteristics of each inhibitor experimentally measured against human mitochondria MetRS, *Ec*MetRS, human liver hepatocellular (Hep G2) and lymphocytic (CRL-8155) cell lines are also shown.

Previous studies have shown good correlations between binding affinities predicted by thermal shift and direct measurement of compound activity [36]. Preliminary determination of *Bm*MetRS and *E*. *coli* MetRS (*Ec*MetRS) IC_50_ values were then performed using a non-radioactive readout, Kinase-Glo^®^ (Promega, Madison, WI) which measures changes in initial ATP concentration using enzyme concentration of 50 nM in the reaction mixture. The sensitivity of this preliminary ATP-depletion assay was optimized based on consumption of around 85% [37] of initial concentration of 1 μM. This ATP concentration is lower than the K_m_ of 85 μM previously reported for a similar human mitochondrial MetRS or K_m_ of 20 μM from *Ec*MetRS [37, 38]. Using the ATP-depletion assay, 50 nM of enzyme gave the best signal range for IC_50_ determination which is consistent with previous findings [37]. The IC_50_ values for the inhibitors tested by this method were found to be 302 nM, 2,887 nM and 7,369 nM for **1312**, **1433** and **1415** respectively (Fig 2). Finally using the aminoacylation enzyme inhibition assay, direct measurement of [^3^H]-L-methionine incorporation into charged aminoacylated tRNA by *Bm*MetRS (2 nM final concentration) in the presence of inhibitors was determined. Compound **1312** IC_50_ value for *Bm*MetRS determined using the aminoacylation assay was 6 nM compared to 272 nM for **1433** and 1657 nM for **1415** (Fig 2).

### *B*. *melitensis* growth inhibition

Growth inhibitory effects on *B*. *melitensis* by MetRS inhibitors were initially assessed based on compound IC_50_ values for *Bm*MetRS. The assay of compound inhibition of bacterial growth was performed using different quantity levels of compound concentrations: **1312**: low (0.376 μg/mL), medium (3.76 μg/mL) and high (37.6 μg/mL); **1415** low (0.716 μg/mL), medium (7.16 μg/mL) and high (71.6 μg/mL); and **1433** low (0.696 μg/mL), medium (6.96 μg/mL) and high (69.6 μg/mL) (Table 1). Inhibition of *B*. *melitensis* growth by MetRS inhibitors was shown to be concentration dependent (Table 1).

**Table 1:** OD_450_ counts and growth inhibitory effects on *B*. *melitensis* exposed to MetRS inhibitors.

	OD 450nm				
Compound name/concentration	1433	1415	1312	Gentamycin (50μg/ml)	No compound
**High**	0.116	0.124	0.13	0.12±0.002	0.4507±0.0111
**Medium**	0.235	0.386	0.34		
**Low**	0.432	0.45	0.45		

Compound **1312** have very efficient (≥95%) inhibition at a concentration of 37.6 μg/mL with identical OD_450_ nm reading as gentamicin at 50 μg/mL, while equivalent level of bacterial growth inhibition was observed in **1415** and **1433** at 69.6 and 71.6 μg/mL respectively. Very little inhibition of *Brucella melitensis* growth was observed at the lowest concentration of inhibitors corresponding to no inhibitor control for all three inhibitors. The minimum inhibitory concentration (MIC) is the lowest concentration of an antimicrobial that will inhibit the visible growth of a microorganism after overnight incubation. The MIC measurements were assessed from growth inhibition values observed over concentrations ranging from 0.696–71.6 μg/mL of inhibitors (Fig 2). The resulting MIC values calculated using Graphpad^®^ Prism software (GraphPad Software, San Diego, CA) were similar for all inhibitors (Fig 2). In the case of the **1433** inhibitor the MIC is 38.67 μg/mL, for **1415** the MIC is 53.93 μg/mL and for **1312**, it is 28.96 μg/mL.

### Structure of *Bm*MetRS bound to substrate analog SeMet*-*

**Fig 3:**
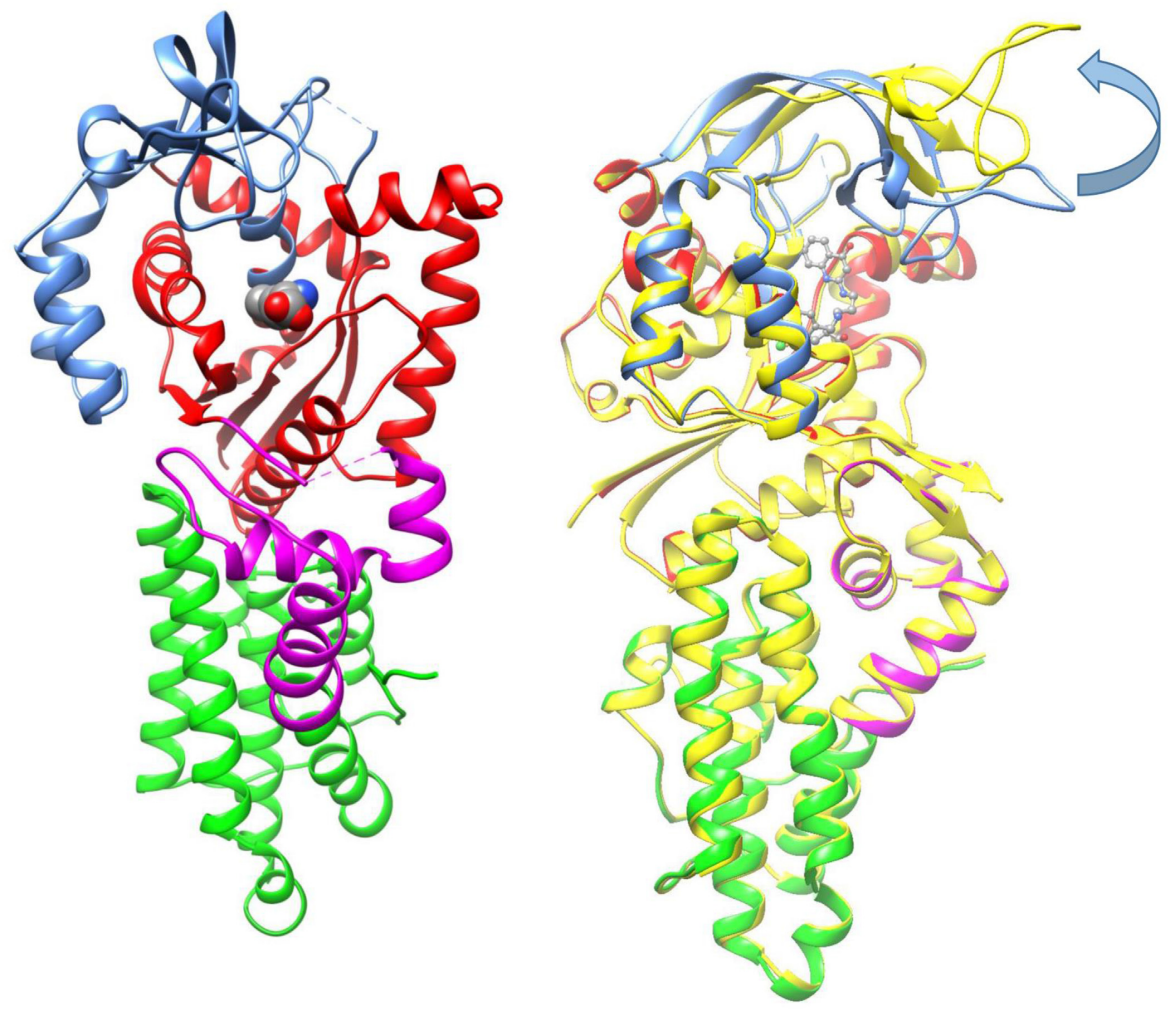
Structures of MetRS: **A. MetRS bound to SeMet.** Structural features of *Bm*MetRS include a catalytic domain formed by a Rossmann fold (red), inserted connective peptide (CP) zinc finger domain (blue), a stem-contact fold (SCF) domain (magenta), and an anti-codon binding α-helix bundle (green). **B. Overlay of MetRS bound to SeMet and MetRS bound to 1312.** MetRS bound to **1312** is colored in yellow. Domains in MetRS bound to SeMet are colored identically to Fig 3a above. Movement of the CP domain is highlighted by the arrow.

*Bm*MetRS incubated with the substrate analog selenomethionine (SeMet) crystallized into space group *P*2_1_ and diffracted to 2.65 Å with three copies in the asymmetric unit. Each copy is similar to each other with RMSDs of 0.39 Å between chain A and B, 0.42 Å between chains A and C, and 0.23 Å between chains B and C. Data collection and refinement statistics are presented in Table 2. *Bm*MetRS is characterized by the structural motifs typically found in MetRSs from other organisms: a Rossman fold catalytic domain (red), inserted connective peptide (CP) domain (blue), a stem-contact fold (SCF) domain (magenta), and an anti-codon binding α-helix bundle (green) (Fig 3a and 3b).

**Table 2:** Data collection and model refinement statistics.

Crystal	SeMet	1312	1415	1433
PDB code	4DLP	5K0S	5K0T	4PLY2
Data collection				
Space Group	P 1 21 1	P 1	P 1	P 1
Cell dimensions				
*a*, *b*, *c* (Å)	116.25, 77.62, 116.27	45.27, 99.74, 104.63	45.16, 99.65, 104.30	45.01, 99.48, 104.00
Α, β, γ (°)	90, 119.67, 90	110.58, 87.63, 99.91	110.46, 88.09, 99.72	110.47, 87.24, 99.99
Resolution range (Å)	50.00–2.65 (2.72–2.65)	50.00–2.40 (2.46–2.40)	50.00–2.60 (2.67–2.60)	50.00–2.15 (2.21–2.15)
No. of unique reflections	52379	63652	49660	88924
R_merge_ (%)^a^	7.1 (48.6)	6.4 (52.5)	7.5 (47.0)	6.6 (53.6)
Redudancy ^a^	3.7 (3.7)	2.4 (2.4)	2.4 (2.4)	3.9 (4.0)
Completeness (%)^a^	99.3 (99.5)	96.3 (85.2)	96.0 (97.5)	98.2 (97.3)
l/σI^a^	15.21 (2.63)	11.43 (2.13)	10.12 (2.07)	15.76 (2.73)
Refinement				
Resolution range	50.00–2.65 (2.72–2.65)	50.00–2.40 (2.46–2.40)	50.00–2.60 (2.67–2.60)	50.00–2.15 (2.21–2.15)
No. of protein atoms	10934	11469	11189	11480
No. of water molecules	88	219	169	135
*R*_cryst_ (%)	19.7	21.0	25.6	17.8
*R*_free_ (%)	23.7	25.6	28.6	21.1
Root-mean-square deviations from ideal stereochemistry				
Bond lengths (Å)	0.012	0.003	0.008	0.011
Bond angles (°)	1.421	0.577	1.183	1.384
Mean B factor (all atoms) (Å^2^)	48.25	43.29	49.76	35.98
Ramachandran plot				
Favored region (%)	96.87	98.77	98.67	97.43
Allowed regions (%)	3.13	1.23	1.33	2.57
Outlier regions (%)	0.00	0.00	0.00	0.00
Clashscore	2.3	2.5	0.46	0.79
Molprobity Score	1.18	1.15	0.74	0.75

^a^ Values in parentheses are for highest-resolution shell

**Fig 4:**
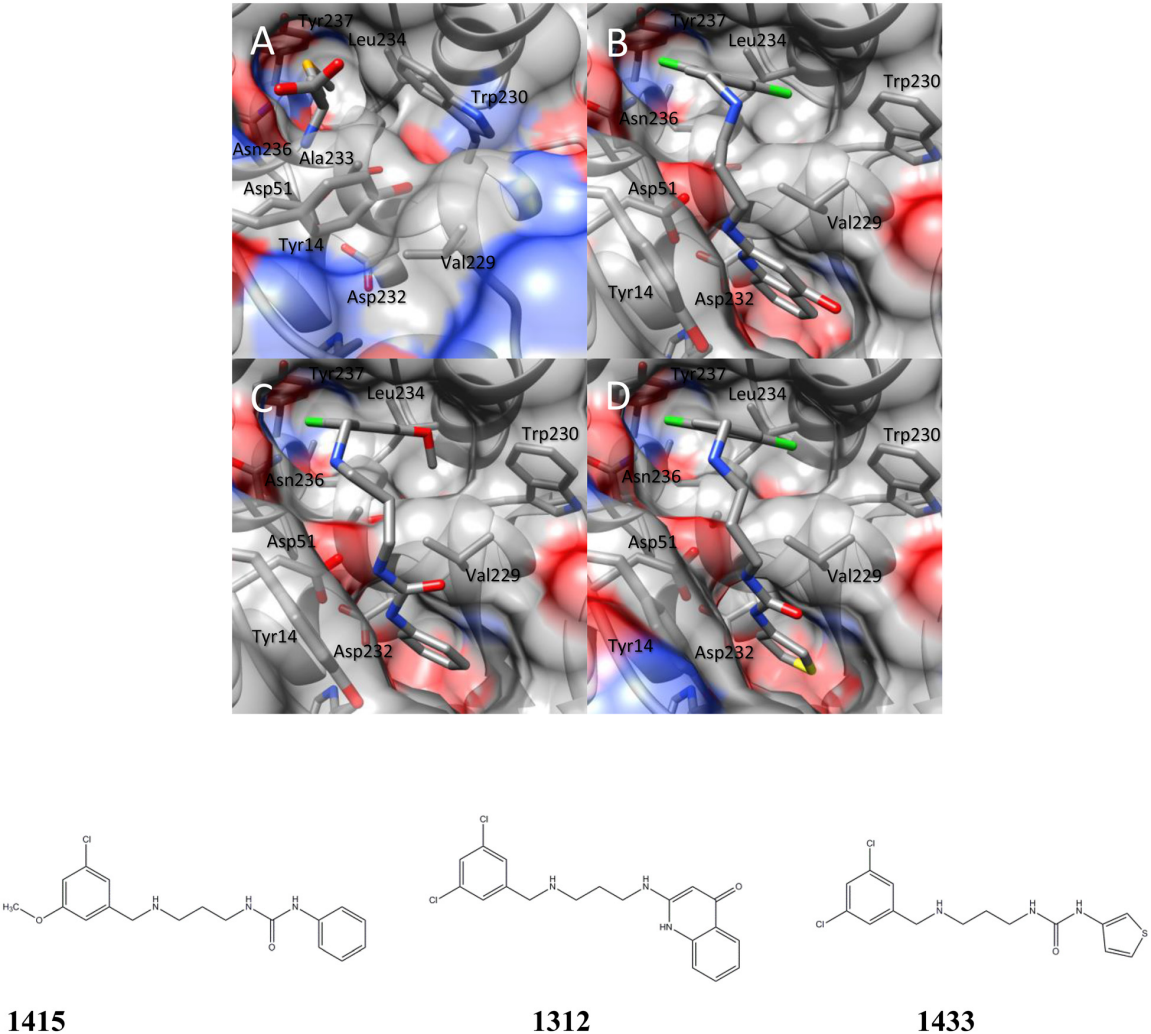
Crystal structure of *Bm*MetRS in-complex with selenomethione and three different inhibitors to define structural pockets. The binding of each ligand; selenomethionine (Fig 4a), **1312** (Fig 4b), **1415** (Fig 4c) and **1433** (Fig 4d) in the crystal structure at the active site causes similar conformational changes to the residues surrounding the methionine-binding site.

Clear electron density for SeMet was observed in each copy of *Bm*MetRS, and anomalous difference density for the selenium atom was present above the 15 σ contour level in all three copies of the protein in the asymmetric unit. SeMet binds in the pocket formed by Tyr14, Asp51, Val229, Trp230, Ala233, Leu234, Asn236, and Tyr237. It makes hydrogen bonds with the side chain of Asp51 and the main chain carbonyl of Ile12 (Fig 4a).

### *Bm*MetRS in complex with inhibitors

**Fig 5:**
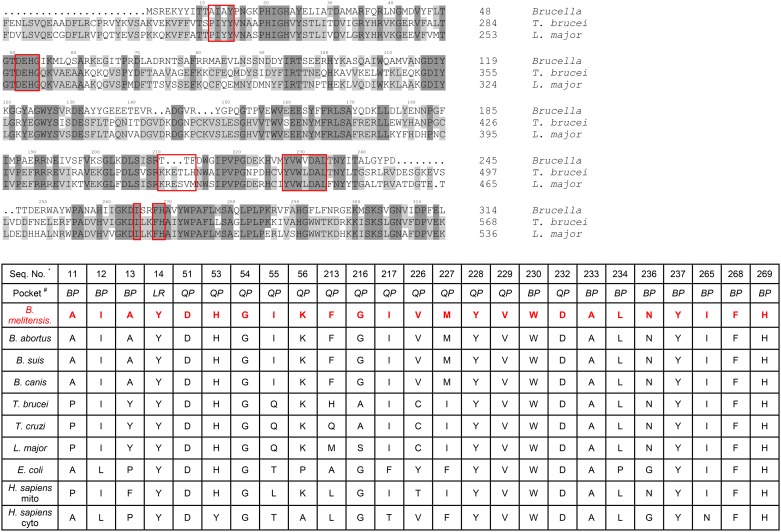
Amino acid sequence alignments within the benzyl and quinolone pocket of *Bm*MetRS, *Tb*MetRS and *Lm*MetRS. Inhibitor binding residues in *Bm*MetRS compared to *Tb*MetRS and *Lm*MetRS are highlighted in red boxes. The most significant differences can be found in residues 211–213 (TTF) of the *Bm*MetRS which are analogous to a larger run of residues (*Tb*MetRS: KRETLH *Lm*MetRS: KRESVM) in the trypanosome structures. Inhibitor interaction with Phe213 within the *Bm*MetRS complex led to different protein geometry relative to the *Tb*MetRS complex. Table shows list of residues within the benzyl and quinolone pockets interacting with inhibitors relative to other MetRS. Sequence numbers refer to the *Bm*MetRS sequence. # LR = linker region, BP = benzyl pocket, QP = quinolone pocket.

In the presence of inhibitors, *Bm*MetRS crystallized in *P*1 with nearly identical unit cell dimensions despite differing crystallization conditions. In each structure there were three copies in the asymmetric unit that were roughly equivalent to each other with average internal RMSDs of 0.81, 0.35, 0.64 for **1312**, **1415**, and **1433** respectively (Fig 4b–4d). Binding of each inhibitor causes similar conformational changes to the residues surrounding the methionine-binding site (benzyl pocket Fig 5). Although the changes are similar in the presence of all three molecules, they are different relative to the conformational pose observed in the presence of substrate analog selenomethionine. Tyr14, which in the SeMet-bound structure forms one face of the binding pocket, swings ~50° to open up the binding pocket deeper into the Rossman fold domain. This allows for Asp51 to make hydrogen bonds to the “urea-core” of each compound, coordinating the tail end into the newly opened pocket (quinolone pocket Fig 5). With the tail end coordinated into the linker region, the head fits into the methionine-binding site. To accommodate each substituted phenyl groups of the inhibitors, Trp230 rotates out and away to create the necessary space (Fig 4a–4d).

**Fig 6:**
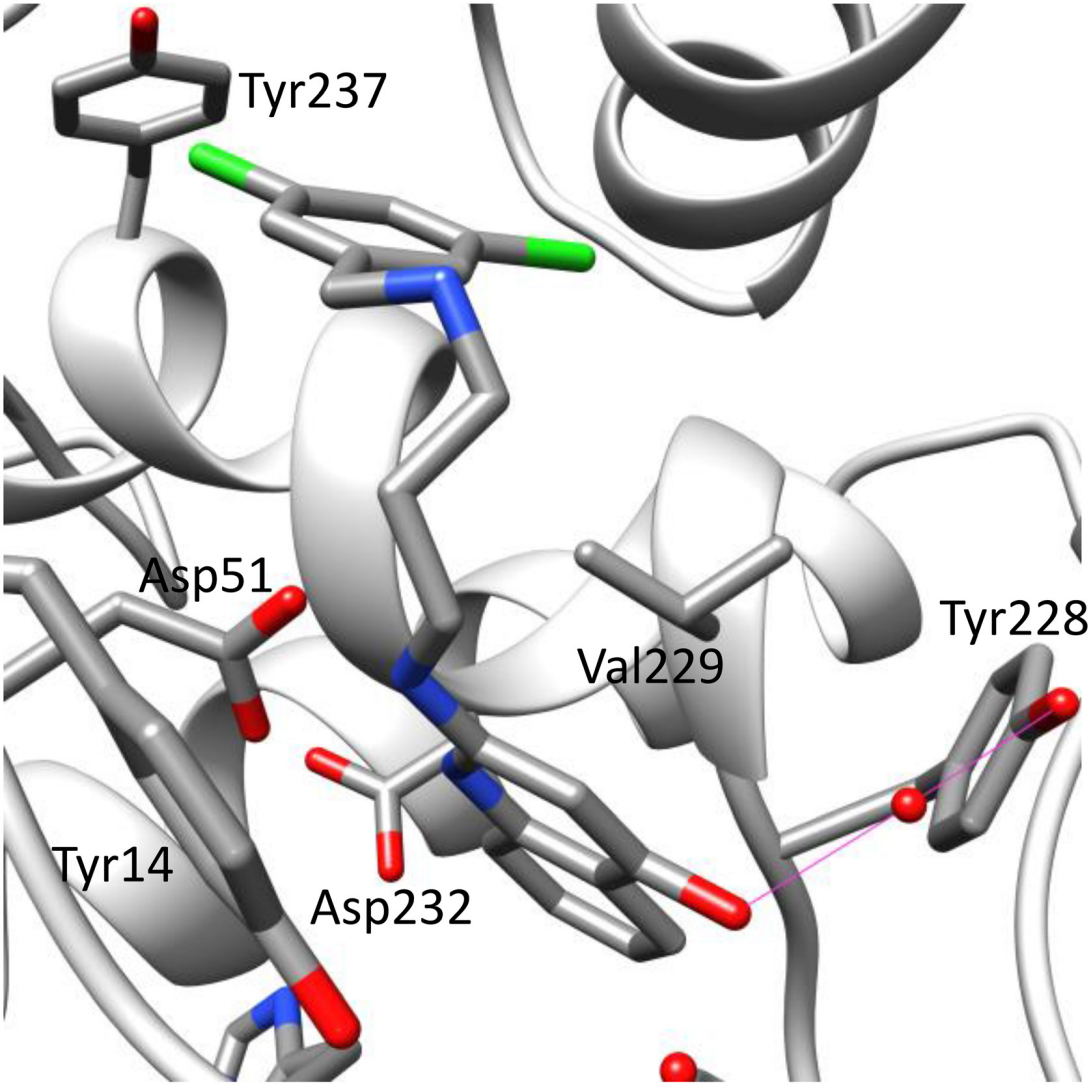
Compound 1312 additional hydrogen bonding of its ketone group with ASP51. The crucial hydrogen bonds in all 3 inhibitors with Asp51 are conserved in all *Bm*MetRS inhibitor complexes. In addition, the 4-ketone group in the aminoquinolone compound **1312** makes water-mediated interactions (water molecule is shown as red sphere) with the phenol hydroxyl of Tyr228.

Based on analysis of the crystal complexes, compound **1312**’s more potent inhibition of *Bm*MetRS relative to the other 2 compounds may be due to the additional hydrogen bonding made by its ketone group with Asp51 in addition to a water mediated interaction with Tyr228 (Fig 6).

**Fig 7:**
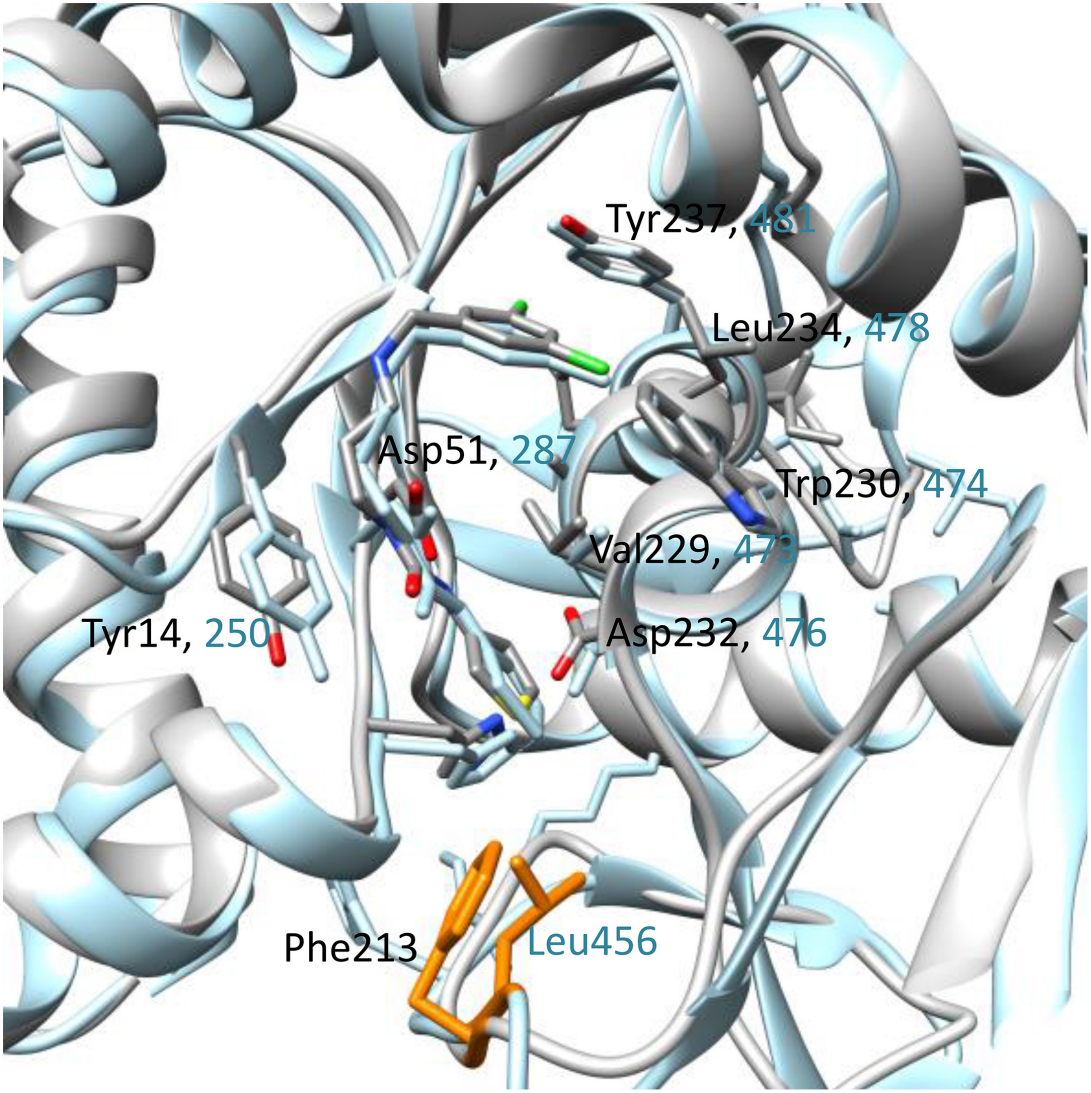
A superposition of *Bm*MetRS (PDB ID 4PLY2 Chain B) and *Tb*MetRS (PDB ID 4MVW Chain) bound to compound 1433. A key difference is the interaction of *Bm*MetRS Phe213 which is functionally equivalent to Leu456 in *Tb*MetRS but led to different protein geometry. The *Tb*MetRS structure is shown in blue and the 2 different residues in orange.

In comparison, there are 6 amino acid differences in the aminoquinolone-based inhibitor’s binding pockets of *Bm*MetRS and *Tb*MetRS (Fig 5). Theoretically, they should be mostly inconsequential as a direct visualization of the superposition of the 2 structures would suggest that these 6 side chains should be pointing away from the pocket. This is especially true when we compare the selenomethionine bound (PDB 4DLP) *Bm*MetRS crystal structure to that of *Tb*MetRS, because the methionine substrate bound conformation is different from the inhibitor bound conformation as observed with *Tb*MetRS. However, there is one region of the inhibitor binding site that stands out. Residues 211–213 (TTF) of the *Bm*MetRS are analogous to a larger run of residues (*Tb*MetRS: KRETLH *Lm*MetRS: KRESVM) in the trypanosome structures (red box in sequence alignment in Fig 5). Because of this, the interaction of Phe213 with the inhibitor seen in the *Brucella* complex instead involves a different residue (Leu456) and a different protein geometry than in the *Tb*MetRS complex. A superposition of 4PLY2 Chain B and 4MVW Chain A gives a good illustration of this (Fig 7). Previous studies have shown that there are dramatically different responses of crystals to the 2 sub-units of aminoquinolone and urea-based inhibitors [21] hence, it is not informative to compare the exact shapes of the inhibitor binding pocket of various MetRS species. Therefore, understanding of inhibitor affinity within *Bm*MetRS pockets through comparative analysis of the structural-activity relationships (SAR) and computational modeling of X-ray crystal structures in complex with inhibitors can effectively guide optimization for selectivity and subsequently improve pharmacokinetic properties.

## Discussion

**Fig 8:**
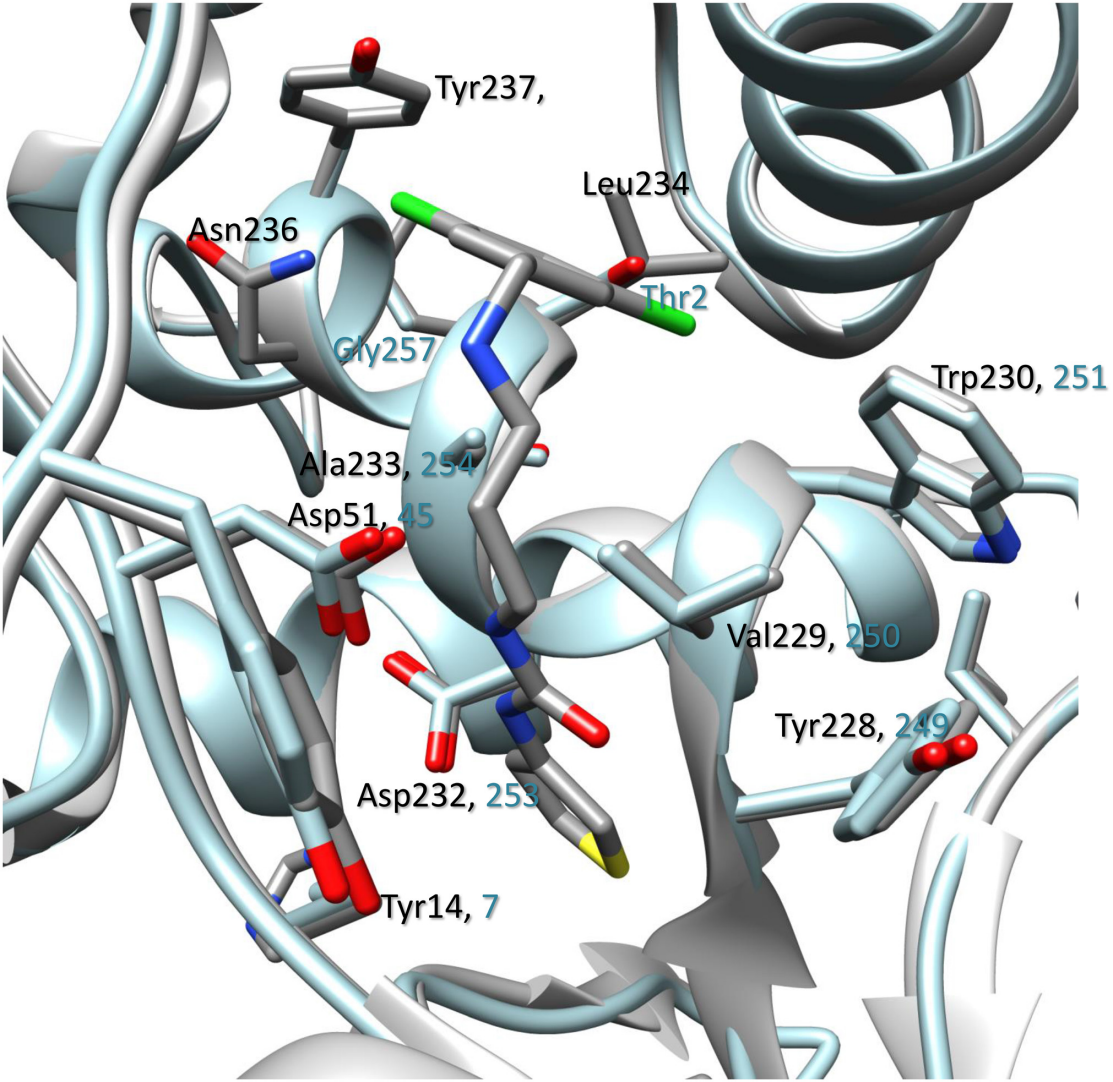
Homology model of human mitochondrial MetRS overlaid with the crystal structure of *Bm*MetRS in complex with 1433. Very few differences could be observed between amino acid residues within the inhibitor binding site and interaction of the compound in the 2 enzymes. This fits well with experimental data showing similar level of inhibition between *Bm*MetRS and human mitochondrial MetRS enzymes.

Therapeutic challenges associated with human brucellosis are multifaceted. Treatment regimens normally include two or more antimicrobials, yet the most effective combination and/or treatment durations are still very ambiguous [39, 40]. Varying rates of therapeutic relapses even with prolonged treatment have been reported for most antimicrobial regimens [41]. Relapses to treatment regimens are thought to be associated with the intracellular localization and replication of *Brucella* species in mammalian hosts. Combating intracellular persistence requires prolonged duration of drug treatment, which may lead to the development of acquired drug resistance not only to the targeted *Brucella* spp. but other human pathogens that respond to similar antimicrobial agents, such as *Mycobacterium tuberculosis*. The prevalence of human brucellosis was earlier shown to increase with age in rural communities and with low socio-economic status [4, 42] similar to tuberculosis. Hence, there is public health concern for a collateral increase of antimicrobial resistance in unrecognized tuberculosis infections with brucellosis therapy, due to overlaps in treatment regimen (streptomycin and rifampin) and prolonged durations of use [6]. Human brucellosis presents clinicians and research scientists’ significant challenges that have resulted in appeals for shorter duration of current treatment regimens and development of more effective novel alternative drug options. It is anticipated that suppression of MetRS would be a major disruption to *Brucella* due to inhibition of initiation and elongation of protein synthesis. Effectively halting protein synthesis should shut down secretion of *Brucella* proteins that are necessary to influence host cells, such as preventing infected host cells from undergoing apoptosis. The aminoquinolone- and urea-based compounds in this study represent a novel class of antimicrobial agents whose synthesis was based on earlier reports of antibacterial activities of MetRS inhibitors [43, 44]. Comparative sequence alignment data (Fig 5) showed that *Bm*MetRS clearly has one knuckle and is in the same MetRS1 family as *Tb*MetRS. Similarly, amino acid residues within the compound binding sites are highly conserved, especially within the benzyl pockets. Hence, huge differences in binding characteristics of *Bm*MetRS and *Tb*MetRS or sensitivity to similar inhibitors are not expected. However, differences in the properties of cell membranes, walls or barriers encountered by chemical entities before gaining access into the cytosolic spaces may influence level of sensitivity of different microorganisms to the same inhibitors. These compounds have shown great therapeutic potential for *Trypanosoma cruzi*, which proliferates as intracellular amastigotes within murine 3T3 fibroblasts, while showing no toxic effect on the mammalian cells [12]. Effective inhibition of *T*. *cruzi* during intracellular growth demonstrates that these inhibitors can act intracellularly [12], overcoming that limitation of brucellosis therapy. Three inhibitors were chosen from a library of one hundred based on a thermal shift stability assay that measures binding to recombinant *Bm*MetRS. The most significant change in thermal shift temperature of 7.8°C was seen for compound **1312**, which also gives the best IC_50_ value against recombinant *Bm*MetRS. The IC_50_s were higher for the luciferase assay than the aminoacylation assay (Fig 1) due to limitations of the Kinase-Glo^®^ assay requiring high ATP and Met levels that effectively compete out aminoquinolone-based inhibitors, while lower ATP/Met levels are required in the aminoacylation assay. The differences in the 3 compounds’ IC_50_s did not translate into appreciably different MIC values in the *B*. *melitensis* growth assay though compound **1312** which has the best inhibitory effect on the target enzyme looks like the best candidate after MIC determination (Fig 1). Since the MetRS inhibitors used in this study were not designed to compete with ATP for binding to the enzyme target, we do not anticipate that the K_m_ of ATP would have any serious effect or could have affected the outcome of the MIC determination. It could however be due to limitations in permeability across the gram-negative membrane by the compound that is more active on the enzyme compared with compounds that are less active on the target enzyme. We have earlier described the poor membrane permeability of **1312** and the optimization strategy of replacing its aminoquinolone group with urea to develop compound **1433** [20, 21]. This led to a considerable gain in cell permeability of **1433** relative to **1312** [20, 21] which may explain the nonlinear co-relationship between enzymatic and cell based responses observed between the two compounds. Similarly, multidrug efflux pumps that exhibit low specificity and confer resistance to several unrelated toxic compounds in varying degree when overexpressed have been reported in *B*. *melitensis* [45]. These compounds, based on their molecular structures, should have limited to no effects on human cytosolic MetRS as earlier demonstrated for similar compounds[17]. However, the IC_50_ values from human mitochondrial MetRS is very similar to that of *Bm*MetRS, especially for the most effective compound **1312**. Similarities in IC_50_ values could be explained with the homology model of the human mitochondrial MetRS overlaid with the crystal structure of *Bm*MetRS in complex with **1433**. Most of the residues within the binding site appear to be well conserved, especially within the benzyl pockets (Figs 5 and 8). Nevertheless, toxicity was not observed in mammalian lymphocytic (CRL-8155) and human liver hepatocellular (Hep G2) cell lines for compound **1312** at concentrations above 20 μM (Fig 1), nor in mice injected with 50 mg/kg of body weight [12, 46]. This suggests the compound probably gets into the cytosol with no inhibitory effects on human cytosolic MetRS, but may have very limited or no interaction with mitochondrial MetRS due to multiple barriers including mitochondrial membrane layers. Other reasons for the absence of toxicity of these compounds in the mouse experimental model or in *in vitro* human cell assays despite potent inhibition of human mitochondrial MetRS have previously been described [12].

## Conclusion

This study presents data showing that specific inhibition of *Bm*MetRS by drug-like molecules could be detrimental to the viability of the bacteria, *Brucella melitensis*. Since only one MetRS has been identified in *Brucella spp*., complementary rescue of essential pathways affected by inhibition of *Bm*MetRS by a second MetRS is very unlikely [15] making this a promising target for brucellosis drug development. Further studies needed to optimize lead compound potency, efficacy and safety as well as determine the pharmacokinetics, optimal dosage, and duration for effective treatment are ongoing. Alterations for optimization will be based on detailed understanding of inhibitor interactions as demonstrated by X-ray crystal structures and iterative improvement with functional groups needed to enhance potency, selectivity, as well as improvement in gram-negative membrane permeability and pharmacokinetic properties. Given the shared mode of action on protein synthesis, we speculate that combination therapy of aminoquinolone-based inhibitors with doxycycline as an alternative treatment for brucellosis will synergize and significantly reduce relapses associated with insufficient therapy.

### Ethics Statement

Human lymphocytic cells (CRL-8155 ATCC, WIL2-NS) and human hepatocellular carcinoma (HEPG2 ATCC_ HB-8065) were purchased from the American Type Culture Collection (Manassas, VA, USA, http://www.atcc.org/products/all/SCRC-1041.aspx).
