# Correction: *Brucella melitensis* Methionyl-tRNA-Synthetase (MetRS), a Potential Drug Target for Brucellosis

**DOI:** 10.1371/journal.pone.0163641

**Published:** 2016-09-22

**Authors:** Kayode K. Ojo, Ranae M. Ranade, Zhongsheng Zhang, David M. Dranow, Janette B. Myers, Ryan Choi, Steve Nakazawa Hewitt, Thomas E. Edwards, Douglas R. Davies, Donald Lorimer, Stephen M. Boyle, Lynn K. Barrett, Frederick S. Buckner, Erkang Fan, Wesley C. Van Voorhis

There is an error in the third-to-last sentence of the “BmMetRS in complex with inhibitors” section. The correct sentence is: A superposition of 4PY2 Chain B and 4MVW Chain A gives a good illustration of this (Fig 7).

There is an error in the caption for Fig 7. Please see the correct Fig 7 caption here.

**Fig 7:**
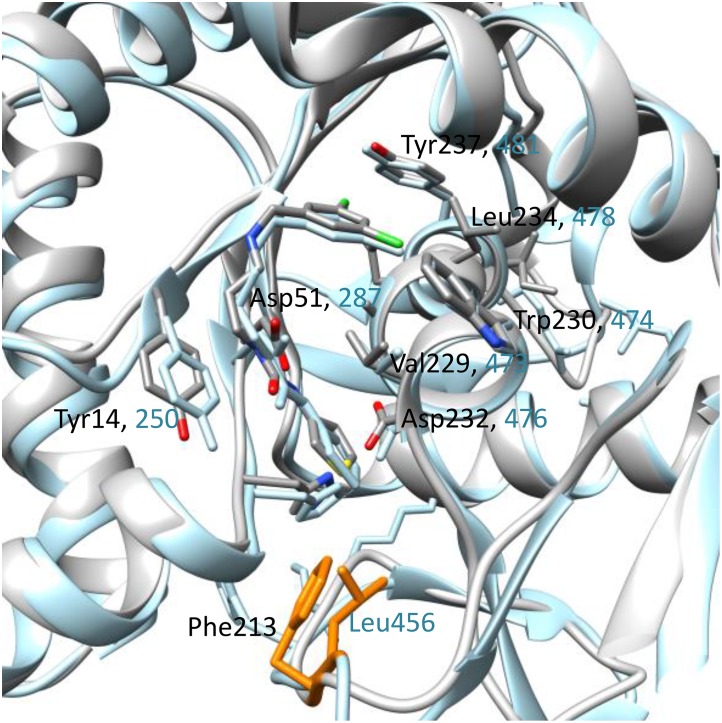
A superposition of *Bm*MetRS (PDB ID 4PY2 Chain B) and *Tb*MetRS (PDB ID 4MVW Chain) bound to compound 1433. A key difference is the interaction of *Bm*MetRS Phe213 which is functionally equivalent to Leu456 in *Tb*MetRS but led to different protein geometry. The *Tb*MetRS structure is shown in blue and the 2 different residues in orange.

Additionally, in Table 2, the “PDB code” value for “1433” should read “4PY2”. Please view the corrected Table 2 here.

**Table 2:** Data collection and model refinement statistics.

Crystal	SeMet	1312	1415	1433
PDB code	4DLP	5K0S	5K0T	4PY2
Data collection				
Space Group	P 1 21 1	P 1	P 1	P 1
Cell dimensions				
*a*, *b*, *c* (Å)	116.25, 77.62, 116.27	45.27, 99.74, 104.63	45.16, 99.65, 104.30	45.01, 99.48, 104.00
Α, β, γ (°)	90, 119.67, 90	110.58, 87.63, 99.91	110.46, 88.09, 99.72	110.47, 87.24, 99.99
Resolution range (Å)	50.00–2.65 (2.72–2.65)	50.00–2.40 (2.46–2.40)	50.00–2.60 (2.67–2.60)	50.00–2.15 (2.21–2.15)
No. of unique reflections	52379	63652	49660	88924
R_merge_ (%)^a^	7.1 (48.6)	6.4 (52.5)	7.5 (47.0)	6.6 (53.6)
Redudancy ^a^	3.7 (3.7)	2.4 (2.4)	2.4 (2.4)	3.9 (4.0)
Completeness (%)^a^	99.3 (99.5)	96.3 (85.2)	96.0 (97.5)	98.2 (97.3)
l/σI^a^	15.21 (2.63)	11.43 (2.13)	10.12 (2.07)	15.76 (2.73)
Refinement				
Resolution range	50.00–2.65 (2.72–2.65)	50.00–2.40 (2.46–2.40)	50.00–2.60 (2.67–2.60)	50.00–2.15 (2.21–2.15)
No. of protein atoms	10934	11469	11189	11480
No. of water molecules	88	219	169	135
*R*_cryst_ (%)	19.7	21.0	25.6	17.8
*R*_free_ (%)	23.7	25.6	28.6	21.1
Root-mean-square deviations from ideal stereochemistry				
Bond lengths (Å)	0.012	0.003	0.008	0.011
Bond angles (°)	1.421	0.577	1.183	1.384
Mean B factor (all atoms) (Å^2^)	48.25	43.29	49.76	35.98
Ramachandran plot				
Favored region (%)	96.87	98.77	98.67	97.43
Allowed regions (%)	3.13	1.23	1.33	2.57
Outlier regions (%)	0.00	0.00	0.00	0.00
Clashscore	2.3	2.5	0.46	0.79
Molprobity Score	1.18	1.15	0.74	0.75
